# Ultrasound Markers for Complex Gastroschisis: A Systematic Review and Meta-Analysis

**DOI:** 10.3390/jcm10225215

**Published:** 2021-11-09

**Authors:** Rui Gilberto Ferreira, Carolina Rodrigues Mendonça, Carolina Leão de Moraes, Fernanda Sardinha de Abreu Tacon, Lelia Luanne Gonçalves Ramos, Natalia Cruz e Melo, Lourenço Sbragia, Waldemar Naves do Amaral, Rodrigo Ruano

**Affiliations:** 1 Postgraduate Program in Health Sciences, Universidade Federal de Goiás, Goiânia 74650-050, GO, Brazil; carol_mendonca85@hotmail.com (C.R.M.); carolina.leao.moraes@gmail.com (C.L.d.M.); fernandabreu2010@yahoo.com.br (F.S.d.A.T.); dr@waldemar.med.br (W.N.d.A.); 2 Department of Obstetrics and Gynaecology, Hospital das Clínicas, Universidade Federal de Goiás, Goiânia 74605-020, GO, Brazil; 3 Hospital das Clínicas, Universidade Federal de Goiás, Goiânia 74605-020, GO, Brazil; leliabiomed@gmail.com; 4 Departamento de Ginecologia, Universidade de São Paulo, São Paulo 04024-002, SP, Brazil; cruz.melo20@gmail.com; 5 Division of Pediatric Surgery, Department of Surgery and Anatomy, Ribeirão Preto Medical School, University of Sao Paulo (USP), Ribeirão Preto 14049-900, SP, Brazil; sbragia@fmrp.usp.br; 6 Division of Maternal-Fetal Medicine, Department of Obstetrics, Gynecology and Reproductive Sciences, University of Texas Health Science Center Houston (UTHealth), Houston 77030, TX, USA

**Keywords:** gastroschisis, prenatal diagnosis, ultrasound, congenital anomalies, fetal surgery, fetal intervention

## Abstract

Although gastroschisis is often diagnosed by prenatal ultrasound, there is still a gap in the literature about which prenatal ultrasound markers can predict complex gastroschisis. This systematic review and meta-analysis aimed to investigate the ultrasound markers that characterize complex gastroschisis. A systematic review of the literature was conducted according to the guidelines of PRISMA. The protocol was registered (PROSPERO ID CRD42020211685). Meta-analysis was displayed graphically on Forest plots, which estimate prevalence rates and risk ratios, with 95% confidence intervals, using STATA version 15.0. The combined prevalence of intestinal complications in fetuses with complex gastroschisis was 27.0%, with a higher prevalence of atresia (about 48%), followed by necrosis (about 25%). The prevalence of deaths in newborns with complex gastroschisis was 15.0%. The predictive ultrasound markers for complex gastroschisis were intraabdominal bowel dilatation (IABD) (RR 3.01, 95% CI 2.22 to 4.07; I^2^ = 15.7%), extra-abdominal bowel dilatation (EABD) (RR 1.55, 95% CI 1.01 to 2.39; I^2^ = 77.1%), and polyhydramnios (RR 3.81, 95% CI 2.09 to 6.95; I^2^ = 0.0%). This review identified that IABD, EABD, and polyhydramnios were considered predictive ultrasound markers for complex gastroschisis. However, evidence regarding gestational age at the time of diagnosis is needed.

## 1. Introduction

Gastroschisis (GS) is an abdominal wall defect diagnosed in prenatal care in more than 90% of cases [1,2]. The diagnosis is usually made by ultrasound in the second trimester of pregnancy to detect floating intestinal loops in the uterine cavity [2]. Gastroschisis can be simple GS or complex GS and the intestinal condition at birth is an important prognostic factor for neonatal comorbidities [3,4]. The two types are differentiated due to the presence of complications in the gastrointestinal area that occurs in complex GS [3].

Complex GS is defined by the presence of congenital intestinal atresia, necrosis, stenosis, perforation, or volvulus [5,6]. Often, more than one complication coexists [5]. Newborns with complex GS stay longer in the hospital, are more likely to be discharged from the hospital with enteral tube feeding and parenteral nutrition, have more morbidities, and mortality is almost 7.6 times higher than in those with simple GS [7].

Although GS is often diagnosed from prenatal ultrasound (US) [8], attempts have been made to correlate US findings with neonatal outcomes in pregnancies with fetal GS [4,9]. However, there is still a gap in the literature about which markers of prenatal US can differentiate complex GS and predict adverse results [10]. Therefore, the objective of this systematic review and meta-analysis is to investigate the ultrasound markers that characterize complex GS and can assist in screening, prenatal counseling, and medical treatment in order to minimize postnatal complications of complex GS.

## 2. Materials and Methods

This systematic review was carried out according to the guidelines of the Preferred Reporting Items for Systematic Reviews and Meta-Analyzes—PRISMA [11] and was registered with the International Prospective Register of Systematic Reviews (PROSPERO) (protocol number: CRD42020211685). No ethical approval or patient consent was required.

### 2.1. Data Sources and Research

The electronic search was carried out in December 2020 in the CINAHL, Embase, and MEDLINE/PubMed databases. Reference lists of eligible studies were also searched, and authors were contacted to obtain unpublished data. The search terms were: (Gastroschisis OR Complex Gastroschisis OR Vanishing gastroschisis) AND (Ultrasound Markers OR Markers ultrasonography OR Sonographic Markers).

All stages of screening the articles were carried out using the Rayyan software [12], which allows a quick exploration and filtering of the eligible studies. The analysis of titles and abstracts was carried out by two researchers independently and the disagreements were resolved by a third researcher. The full reading was performed by two researchers independently. The research was limited to studies carried out in humans.

The criteria to include the patients and studies in the present systematic review were: (1) pregnant women in any gestational week; (2) fetuses with an ultrasound diagnosis of complex GS; (3) studies that reported on ultrasound markers to detect structural anomalies; (4) observational and intervention studies; (5) articles in English; (6) no restriction regarding the year of publication. The presence of intestinal atresia, stenosis, volvulus, necrosis, or intestinal perforation at birth was defined as complex GS [6]. The exclusion criteria were as follows: the use of markers other than ultrasound, studies that did not differentiate simple GS from complex GS in the results of ultrasound markers, case reports or reviews on the diagnosis of complex GS, conference abstracts, experimental research, or in vitro studies.

After reading the studies (manuscripts) in full, the following data were collected: authors and year of publication, study design, country where the study was conducted, sample size, age, gestational age at the time of delivery, ultrasound markers, and outcomes. The variables investigated for ultrasound markers were intraabdominal bowel dilatation (IABD), extra-abdominal bowel dilatation (EABD), intrauterine growth restriction, polyhydramnios, intestinal wall thickness, bowel dilatation, liver and bladder herniation, delta dilatation and final bowel dilatation, abdominal circumference, herniation, dilation of the stomach, size, and position of stomach, size of the abdominal wall defect, description of mesenteric circulation, collapsed extra-abdominal bowel, description of peristalsis and volvulus.

### 2.2. Bias Risk and Quality Assessment

The risk of bias assessment was analyzed using the tool “A Cochrane Risk of Bias Assessment Tool for Non-Randomized Studies” [13] using the ROBINS-I software [14]. Eight methodological domains were evaluated: (1) bias due to confounding, (2) bias in selection of participants into the study, (3) bias in measurement of interventions, (4) bias due to departures from intended interventions, (5) bias due to missing data, (6) bias in measurement of outcomes, (7) bias in selection of the reported result, and (8) overall bias. Each domain was assigned a “low risk of bias”, “moderate risk of bias”, “serious risk of bias”, and “critical risk of bias” judgment.

The quality of the studies was assessed using the Grading of Recommendations, Assessment, Development, and Evaluations (GRADE) [15]. The quality of the study’s evidence was classified into four categories: high, moderate, low, or very low [15,16].

### 2.3. Statistical Analysis

Meta-analysis was conducted using the random-effects model on coded data stratified by complex GS characteristics, mortality rate, complex GS ultrasound markers, and comparison of ultrasound markers in SCG and complex GS. The data were displayed graphically in Forest plots, which estimate prevalence rates and risk ratios, with 95% confidence intervals (CI). The statistical values I^2^ were calculated to quantify the degree of heterogeneity between studies, where values of 25–50% represented moderate heterogeneity and values of >50% great heterogeneity between studies [17]. Publication bias was assessed using the Egger test. All analyzes were conducted using STATA version 15.0 (StataCorp, College Station, TX, USA).

## 3. Results

### 3.1. Search Results

**Figure 1:**
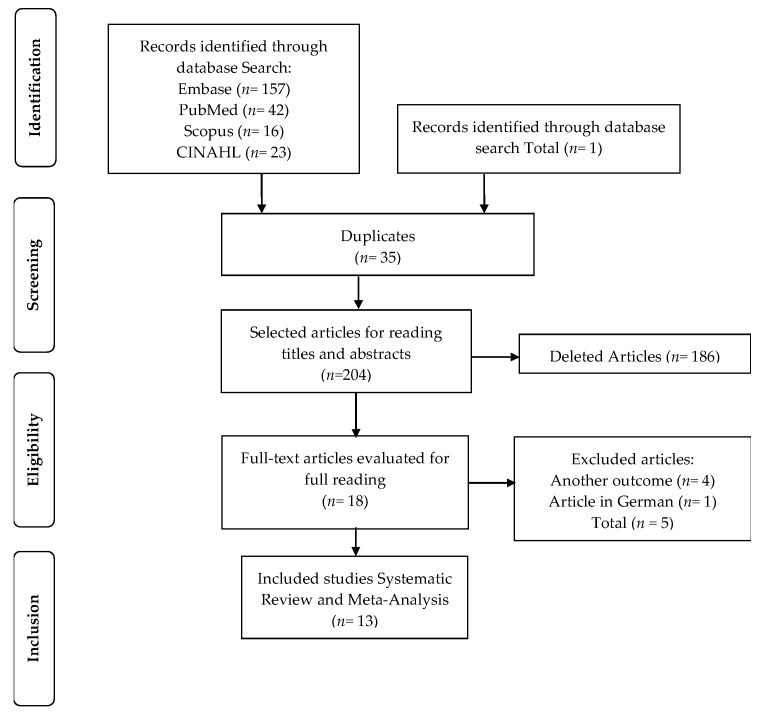
Flowchart of study selection.

The initial search identified 238 articles. After excluding duplicate articles (*n* = 35), the titles and abstracts of 204 articles were read. Of these, 18 were selected for full reading. A total of 13 articles met the inclusion criteria [1,3,6,8,9,18,19,20,21,22,23,24,25]. The study selection flowchart is shown in Figure 1.

### 3.2. General Characteristics

The 13 studies that met the inclusion criteria involved a total of 1440 fetuses with GS, with 274 fetuses (19.02%) with complex GS. The average weight of fetuses with complex GS was 2341 g. The average maternal age was 23.8 years, and the average gestational age at delivery was 35.5 weeks. Details on the characteristics of the studies are presented in Table 1 and Table S1.

### 3.3. Assessment of Quality and Risk of Bias

**Figure 2:**
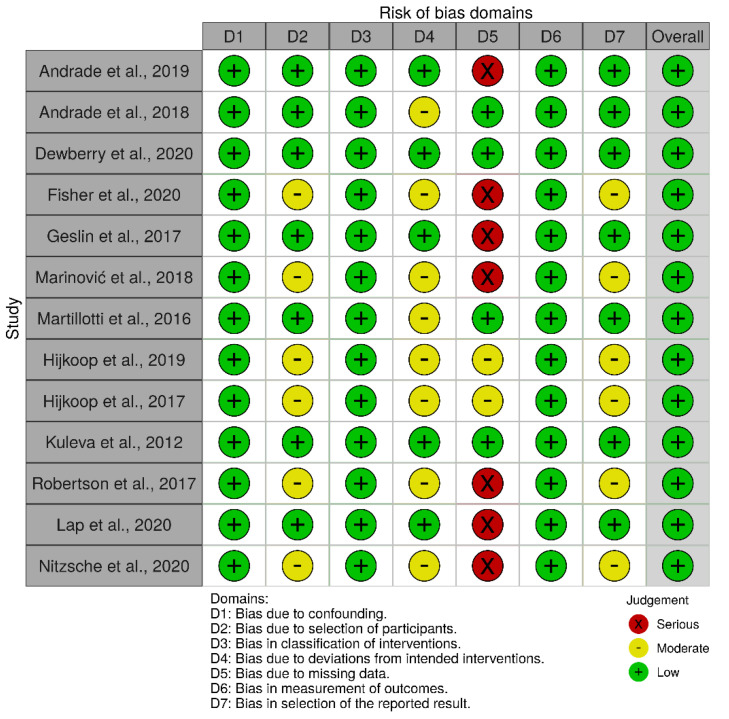
Assessment of the risk of bias.

A total of 12 cohort studies and a case-control study were assessed using the GRADE quality assessment tool (Table 1) and risk of bias by the Cochrane tool for non-randomized studies (Figure 2). The GRADE score indicated that five studies showed low quality of evidence [1,3,8,22,24] and eight studies with moderate quality of evidence [6,9,18,19,20,21,23,25].

The results of the risk of bias assessment of the included studies are shown in Figure 2. Although the risk of bias in general was considered moderate to low, in some studies we identified a serious risk of bias, as the studies did not meet the bias criterion due to missing data. The assessment of quality and risk of bias was influenced by the lack of information and the small sample size.

### 3.4. Ultrasound Markers for Complex Gastroschisis

Data on the definition of complex GS, scan, and ultrasound markers are shown in Table S1. Eight studies reported that IABD measurement is useful in predicting complex GS [6,9,18,19,20,21,23,25]. Four studies reported that the presence of EABD proved to be statistically significant in predicting complex GS [3,9,19,24]. Two studies indicated that the presence of polyhydramnios was shown to be statistically significant in predicting complex GS [8,19]. Two studies reported that US markers could not reliably distinguish between simple GS and complex GS [1,22].

### 3.5. Meta-Analysis

**Figure 3:**
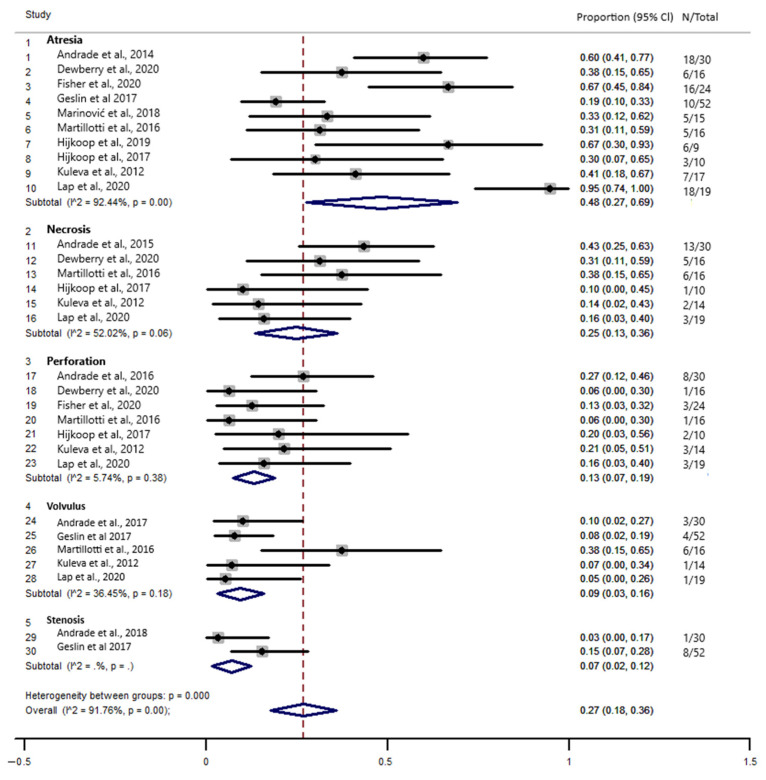
Forest plot of the combined prevalence of atresia, necrosis, perforation, volvulus, and stenosis in fetal complex gastroschisis.

Figure 3 shows the combined prevalence of intestinal complications including atresia, necrosis, perforation, volvulus, and stenosis that are predictors for complex gastroschisis. The combined prevalence was 27.0% (95% confidence interval (CI), 0.18–0.36). Statistical heterogeneity was high (I^2^ = 91.76%, *p* < 0.000). Thus, we performed a meta-regression analysis (tau^2^ = 21.49, I^2^ = 91.38%, Adj R-squared = 11.44%). The analysis showed that heterogeneity had an influence on the analysis result. Using Egger’s regression test, we found evidence of publication bias in the meta-analysis of the combined prevalence of atresia, necrosis, perforation, volvulus, and stenosis (*p* = 0.044).

**Figure 4:**
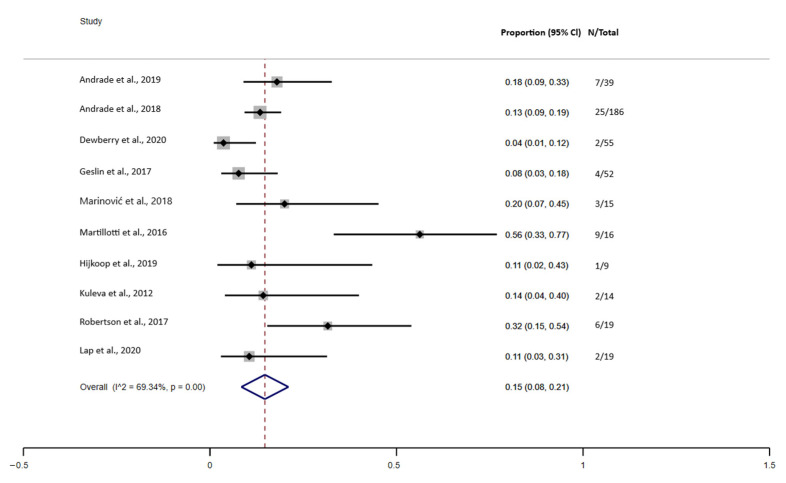
Forest plot of the prevalence of mortality in complex gastroschisis.

Figure 4 indicates a prevalence of 15.0% (95% confidence interval (CI), 0.08–0.21) of deaths in newborns with complex GS. Statistical heterogeneity was high (I^2^ = 69.34%, *p* = 0.00). Therefore, we performed a meta-regression analysis (tau^2^ = 0, I^2^ = 0.00%). The analysis showed that heterogeneity had no influence on the result of the analysis. Using Egger’s regression test, we found no evidence of publication bias in the meta-analysis of the prevalence of mortality from complex GS (*p* = 0.520).

### 3.6. Fetal Ultrasound Evaluation

**Figure 5:**
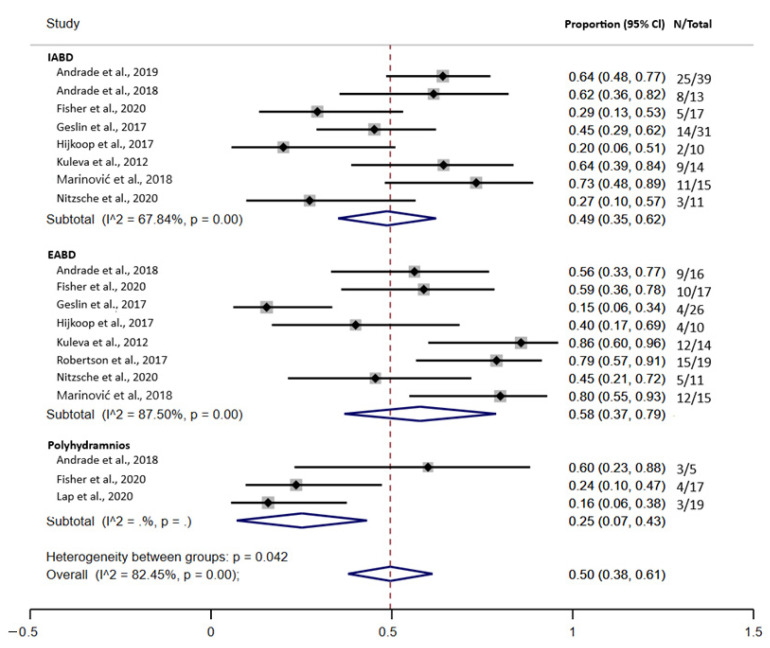
Forest plot of the prediction of complex gastroschisis with intraabdominal bowel dilatation (IABD), extra-abdominal bowel dilatation (EABD), and polyhydramnios.

Figure 5 indicates the combined prevalence of prediction of complex GS with intraabdominal bowel dilatation (IABD), extra-abdominal bowel dilatation (EABD), and polyhydramnios. The meta-analysis indicated that the combined prevalence of ultrasound predictors for complex GS was 50.0% (95% confidence interval (CI), 0.38–0.61). There was a higher prevalence of the EABD ultrasound marker with a prevalence of 58.0% (95% confidence interval (CI), 0.37–0.79), followed by a 49.0% IABD (95% confidence interval (CI), 0.35–0.62) and polyhydramnios was 25.0% (95% confidence interval (CI), 0.07–0.43). The statistical heterogeneity was substantial (I^2^ = 82.45%, *p* = 0.00). The meta-regression showed that heterogeneity had an influence on the results of the analysis (tau^2^ = 13.42, I^2^ = 85.26%, Adj R-squared = 57.61%).

**Figure 6:**
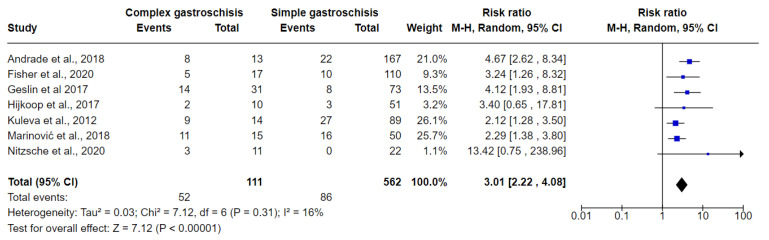
Forest plot between simple and complex gastroschisis for IABD ultrasound markers.

**Figure 7:**
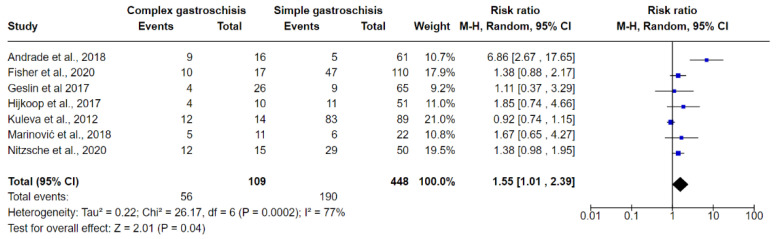
Forest plot between simple and complex gastroschisis for EABD ultrasound markers.

**Figure 8:**
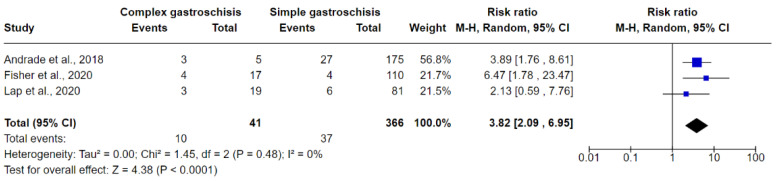
Forest plot between simple and complex gastroschisis for polyhydramnios ultrasound markers.

Figure 6, Figure 7 and Figure 8 show the results of comparisons between complex GS and simple GS for the ultrasound markers IABD, EABD, and polyhydramnios, respectively.

#### 3.6.1. IABD

Seven studies were included in the meta-analysis comparing the use of the IABD ultrasound marker in fetuses with complex GS and simple GS. In total, 52/111 (46.84%) fetuses with complex GS had IABD while 86/562 (15.30%) fetuses with simple GS had IABD. The meta-analysis indicated that the risk of predicting IABD is higher in fetuses with complex GS (RR 3.01, 95% CI 2.22 to 4.08; I^2^ = 16%, *p* = 0.310). The non-significance of the heterogeneity test suggests that the differences between the studies are explained by random variation. Using Egger’s regression test, we found no evidence of publication bias in the meta-analysis (*p* = 0.168) (Figure 6).

#### 3.6.2. EABD

Seven studies were included in the meta-analysis evaluating the presence of EABD in prenatal ultrasound examinations in fetuses with complex GS and simple GS. In total, 56/109 (51.37%) fetuses with complex GS had EABD while 190/448 (42.41%) fetuses with simple GS had EABD. The meta-analysis indicated that the risk of predicting EABD is greater in fetuses with complex GS (RR 1.55, 95% CI 1.01 to 2.39; I^2^ = 77%, *p* = 0.000). The results revealed significant heterogeneity between studies (I^2^ = 77%), so we performed a meta-regression analysis to examine possible sources of heterogeneity. The analysis showed that no heterogeneity and no inconsistency had any influence on the results of the analysis (tau^2^ = 0, I^2^ = 0.00%). Using Egger’s regression test, we found no evidence of publication bias in the meta-analysis (*p* = 0.945) (Figure 7).

#### 3.6.3. Polyhydramnios

Three studies were included in the meta-analysis evaluating the presence of polyhydramnios on ultrasound examination in fetuses with complex GS and simple GS. In total, 10/41 (24.39%) fetuses with complex GS had polyhydramnios while 37/366 (10.10%) fetuses with simple GS had polyhydramnios. The meta-analysis indicated that the risk of predicting polyhydramnios is greater in fetuses with complex GS (RR 3.82, 95% CI 2.09 to 6.95; I^2^ = 0.0%, tau^2^ = 0). Values of I^2^ and Tau ^2^ are consistent with no heterogeneity and no inconsistency (Figure 8).

## 4. Discussion

Here, through systematic review and meta-analysis, we reviewed the evidence available on ultrasound markers that characterize complex gastroschisis. Thirteen cohort and case-control studies carried out in different countries and with moderate to low risk of bias, were included. The ultrasound markers that showed to be statistically significant in predicting complex GS were IABD [6,9,18,19,20,21,23,25], EABD [3,9,19,24], and polyhydramnios [8,19].

Complex GS is known to be associated with greater morbidity and mortality than simple GS. Thus, prenatal prediction of intestinal complications in infants with complex gastroschisis is important to identify cases that may benefit from early obstetric intervention [9]. Bergholz et al. and D’Antonio [7,10] initially explored gastroschisis in systematic review and meta-analysis studies. Bergholz et al. described that infants with complex GS start enteral nutrition later and take longer to complete nutrition and consequently a longer duration of parenteral nutrition. The risk of sepsis, short bowel syndrome, and necrotizing enterocolitis is also greater, as is a longer hospital stay [7]. Furthermore, D’Antonio et al. investigated prenatal risk factors and gastroschisis outcomes. These authors found significant positive associations between IABD and intestinal atresia, polyhydramnios, intestinal atresia, and gastric dilatation, and neonatal death [10].

Other prognostic factors related to mortality in neonates with gastroschisis, from prenatal care to corrective surgery, include inadequate prenatal care, low birth weight, gestational age, severity of intestinal injury, infection, and sepsis [26]. Screening of the severity of the intestinal injury is performed by fetal US in prenatal care and allows early determination of parental counseling and optimal perinatal management [27]. US scans can diagnose gastroschisis as early as 12 weeks of gestation [28]. Fetal magnetic resonance imaging can be a complement to US, providing global and detailed anatomical information, assessing the extent of defects, and also contributing to confirming the diagnosis in doubtful cases [27]. Postnatal surgical management is aimed at reducing herniated viscera and closing the abdominal wall. However, the prognosis depends on the condition of the bowel at birth. Infants with significant intestinal damage at birth are “at risk” of premature death or adverse long-term outcomes [28].

It is important to highlight that although there was an attempt to investigate different markers that could predict complex gastroschisis, US markers that showed to be statistically significant in predicting complex GS were IABD, EABD, and polyhydramnios. Furthermore, in the present study, about 46.84% of fetuses with complex GS and 15.30% of fetuses with simple GS had IABD on ultrasound. Regarding EABD, about 51.37% of fetuses with complex GS and 42.41% of fetuses with simple GS had this US finding. Polyhydramnios was detected via ultrasound in 24.39% of fetuses with complex GS and in 10.10% of fetuses with simple GS.

The meta-analysis also indicates that the combined prevalence of intestinal complications in fetuses with complex GS was 27.0%, particularly with a higher prevalence of atresia (about 48%), followed by necrosis (about 25%) and perforation (about 13%). In addition to the presence of these complications, the prevalence of deaths in newborns with complex GS was 15.0%. We did not identify other meta-analyses that reported the combined prevalence of complications in fetuses with complex GS. However, a meta-analysis reported similar results regarding the mortality rate in newborns with complex GS (16.67%) [7]. Although, it is important to note that there was an important variation in the mean gestational age (GA) at the time of ultrasound reported by these studies, but it generally occurred in pregnancies over 26 weeks. It was not possible to predict the influence of the gestational age at the time of diagnosis in predicting complex GS.

### 4.1. Implications for Practice

US is a great tool in the diagnosis of GS. The presence of complications in fetuses with complex GS includes atresia, necrosis, perforation, volvulus, and stenosis and the predictive ultrasound markers are IABD, EABD, and polyhydramnios.

### 4.2. Implications for Research

Future studies evaluating different US markers (IABD, EABD, intrauterine growth restriction, polyhydramnios, intestinal wall thickness, bowel dilatation, liver, and bladder herniation, delta dilatation and final bowel dilatation, abdominal circumference, herniation, dilation of the stomach, size, and position of stomach, size of the abdominal wall defect, description of mesenteric circulation, collapsed extra-abdominal bowel, description of peristalsis and volvulus) in fetuses with complex GS should report the mean gestational age at the time of US diagnosis to evaluate the impact of the time of the presence of those ultrasound markers in predicting complex GS. Larger, well-designed prospective studies that recruit a representative sample of participants are also still necessary. The role of US as diagnostic and predictor strategies should be evaluated, as well as the incorporation of US markers for the diagnosis of complex GS.

### 4.3. Strengths and Limitations

The strengths of this review include a current, comprehensive, and detailed search according to literature and standardized data extraction and the performance of meta-analysis which can to helpful fundament clinical decisions and prevent severe complications of complex GS. The main limitations of the review were the exclusion of studies in languages other than English [29]. Another limitation concerns the sample size of fetuses with complex GS in each study. However, from evidence from previous studies, we recommend that future studies include a more robust sample of fetuses with complex GS.

## 5. Conclusions

Intraabdominal bowel dilatation, extra-abdominal bowel dilatation, and polyhydramnios were considered predictive US markers of complex gastroschisis. However, in view of the fact that we were unable to identify the gestational age at the time of the diagnosis of these findings, we recommend future studies that assess diagnostic accuracy and include sensitivity and specificity tests.

## Figures and Tables

**Table 1:** Characteristics of studies included in the systematic review.

Author, Year	Country	Study Design	Sample Size	Fetuses Complex Gs (*n*) Birth Weight, G	Gestational Age At Delivery, Weeks	Complex Gastroschisis	Diagnostic	Mean Age Of Mother (Years)	Mortality Rate	Risk Of Bias (GRADE)
Andrade et al., 2019 [18]	UK	Retrospective cohort study January 2005 and December 2018	*n* = 174	*n* = 39 (22.4%) complex GS. 2240 (2041–2678) g	35.7 (34.8–37.0)	NR	Ultrasound	20 (19.0–24.0)	17.9% (7/39)	⨁⨁⨁◯ Moderate
Andrade et al., 2018 [19]	Brazil	Retrospective cohort study January 2005 and December 2015	*n* = 186	*n* = 30 (16.1%) complex GS. 2357 ± 461 g	36.1 ± 1.5	Atresia 18/30 (60%) Necrosis 13/30 (43.3%) Perforation 8/30 (26.6%) Volvulus 3/30 (10%) Stenosis 1/30 (3.3%)	Ultrasound	20.98 ± 4.2	13.4% (25/186)	⨁⨁⨁◯ Moderate
Dewberry et al., 2020 [20]	USA	Retrospective cohort study 2007 to 2017	*n* = 55	*n* = 16 complex GS. 2300 g	36 (35–37)	Atresia 6/16 (37.5%) Necrosis 5/16 (31.25%) Perforation 1/16 (6.25%) Cases of vanishing gastroschisis 3/16 (18.75%)	Ultrasound	21 (19–24)	4% (2/55)	⨁⨁⨁◯ Moderate
Fisher et al., 2020 [8]	Indiana	Retrospective cohort study 2010 to 2018	*n*= 134	*n* = 24 complex GS. 2369.1 ± 685.2 g	NR	Atresia and perforation 3/24 (12.5%) Atresia only 16/24 (66.6%) Perforation only 3/24 (12.5%) Other indications of complex gastroschisis (matted bowel, primary bowel dysfunction) 2/24 (8.33%)	Ultrasound	NR	NR	⨁⨁◯◯ Low
Geslin et al. 2017 [21]	France	Retrospective cohort multicentre study January 2000 to October 2013	*n* = 200	*n* = 52 complex GS. NR	35.3 ± 1.5	Bowel atresia 10/52 (19.23%) Stenosis 8/52 (15.38%) Volvulus 4/52 (7.69%) Ischemia 2/52 (3.84%) Fibrous bands responsible for bowel wall compromise 24/52 (46.15%)	Ultrasound	24.3 ± 5.0	7.7% (4/52)	⨁⨁⨁◯ Moderate
Marinović et al., 2018 [25]	Sérvia	Retrospective cohort study NR	*n* = 65	*n* = 15 (23.7%) Complex GS. 2351.33 ± 633.8 g	36.16 ± 1.4	Bowel atresia = 5/15 (7.69%) Stenosis, Perforation e Necrosis = 9/15 (60%) Gastrosquise de fechamento = 1/15 (6.66%)	Ultrasound	NR	20% (3/15)	⨁⨁⨁◯ Moderate
Martillotti et al., 2016 [23]	Canada	Retrospective cohort study over 11 years January 2000 and January 2011	*n* = 117	*n* = 16 complex GS. 2633 (2272–2782) g	35.6 (32.8–37.3)	Volvulus 6/16 (37.5%) Bowel Atresia 5/16 (31.2%) Bowel necrosis 6/16 (37.5%) Bowel perforation 1/16 (6.2%)	Ultrasound	22.8 (19.7–27.5)	56.3% (9/16)	⨁⨁⨁◯ Moderate
Hijkoop et al., 2019 [1]	The Netherlands	Prospective cohort study June 2010 and April 2015	*n* = 79 fetuses	*n* = 9 complex GS. 2220 (1840–2800) g	35.4 (33.5–37.0)	Intestinal atresia 6/9 (66.66%) Intestinal atresia + perforation 1/9 (11.11%) Intestinal atresia + necrosis 1/9 (11.11%) Intestinal atresia + necrosis + volvulus 1/9 (11.11%)	3D ultrasound	24 (22–29)	11% (1/9)	⨁⨁◯◯ Low
Hijkoop et al., 2018 [22]	The Netherlands	Retrospective cohort analysis 2000 to 2012	*n* = 61	*n* = 10 complex GS. 2385 (2228–2525) g	36.8 (36.4–37.4)	Bowel atresia 3/10 (30%) Intestinal atresia + necrosis 2/10 (20%) Intestinal atresia + perforation 1/10 (10%) Necrosis 1/10 (10%) Necrosis + volvulus 1/10 (10%) Perforation 2/10 (20%)	Ultrasound	30.1 (29.7–31.1)	NR	⨁⨁◯◯ Low
Kuleva et al., 2012 [6]	France	Retrospective case-control study 1999 to 2010	*n* = 103	*n* = 14 complex GS. 2325 (2005–2700) g	35.7 ± 1.5 weeks	Bowel atresia 7/14 (6.5%) Bowel perforation 3/14 (2.9%) Colonic diverticulum 1/14 (1.0%) Bowel necrosis 2/14 (2.9%) Duodenal volvulus 1/14 (0.97%)	Ultrasound	25.7 ± 4.7	14.28% (2/14)	⨁⨁⨁◯ Moderate
Robertson et al., 2017 [24]	Australia	Retrospective cohort analysis January 2000 and June 2013	*n* = 101	*n* = 19 complex GS. NR	35.8 (median = 36.6 range = 24.1–41.1)	NR	Ultrasound	23.9	31.57% (6/19)	⨁⨁◯◯ Low
Lap et al., 2020 [9]	The Netherlands	Prospective cohort 2010 and 2015	*n* = 131	*n* = 19 complex GS. 2372 ± 403 g	36.0 (32.3–37.6)	Atresia 18/19 (94.7%) Antenatal volvulus 1/19 (5.3%) Necrosis 3/19 (15.8%) Perforation 3/19 (15.8%)	Ultrasound using a GE Voluson 730 or E8 (GE Healthcare, Zipf, Austria) ultrasound machine, with a 4–8 MHz transabdominal transducer.	26.9 ± 4.9	10.5% (2/19)	⨁⨁⨁◯ Moderate
Nitzsche et al., 2020 [3]	Germany	Retrospective cohort analysis 2007 and 2017	*n* = 34	*n* = 11 complex GS. 2190 (1370–2985) g	33 + 6 (33 + 0–34 + 5)	NR	Ultrasound	23 (between 17 and 37)	NR	⨁⨁◯◯ Low

GS: gastroschisis; NR: not reported.

## Data Availability

The data presented in this study are available on request from the corresponding author.

## Supplementary Materials

The following are available online at https://www.mdpi.com/article/10.3390/jcm10225215/s1, Table S1: Definition, scan, and ultrasound markers of complex gastroschisis.
