# Supplementary material for: Ultrasound Markers for Complex Gastroschisis: A Systematic Review and Meta-Analysis
Source: J Clin Med. 2021 Nov 9;10(22):5215. doi: 10.3390/jcm10225215 (PMC8619043; doi:10.3390/jcm10225215)
Supplement: Supplementary file 1 [file jcm-10-05215-s001.zip › jcm-1417886-supplementary.pdf]

Table S1. Definition, Scan and ultrasound markers of complex gastroschisis.

AUTHOR, YEAR	Definition of complex gastroschisis	Protocol for fetal gastroschisis	Scan	Prenatal Ultrasound Signs Explored	Ultrasound markers CG	Protocol deliveries
Andrade et al., 2019	Presence of intestinal atresia, stenosis, perforation, necrosis or volvulus<br>Intra-abdominal<br>Extra-abdominal	NR	Ultrasound image of cross section	IABD at both 20–22 and 30–32weeks' gestation	At the IABD cut-off of 7mm at 20–22 weeks, the DR, FPR, PPV and NPV for complex gastroschisis were 61.5%, 6.7%, 72.7% and 89.4%, respectively, and at IABD cut-off of 14mm at 30–32weeks, the respective values were 64.9%, 5.9%, 75.0% and 90.7%.	NR
Andrade et al., 2018	Birth by the pediatric surgeon as the presence of intestinal atresia, stenosis, perforation, necrosis, or volvulus	Follow-up scans every 4 weeks until 28 weeks of gestation, every 2 weeks until 34 weeks of gestation, and weekly until 37 weeks of gestation	Fetal growth, amniotic fluid volume, and umbilical artery Doppler	AC (<5th percentile (shortly before delivery))<br>AWD (size/AC ratio (shortly before delivery))<br>EABD<br>EBWT (>3 mm (shortly before delivery))<br>IABD<br>Stomach herniation<br>Stomach dilatation (shortly before delivery)<br>Oligohydramnios<br>Polyhydramnios	EABD ($p = 0,002$)<br>IABD ($p < 0.001$)<br>Polyhydramnios	Deliveries were scheduled at 37 weeks of gestation
Dewberry et al., 2020	Complex gastroschisis is characterized by gastroschisis associated with intestinal abnormalities such as intestinal atresia, necrosis, volvulus, perforation, or vanishing or closed gastroschisis which is one of the more severe forms of complex gastroschisis.	NR	Prenatal ultrasound	IAID and EAID, thickened bowel wall (bowel wall >3 mm), hyperechoic bowel (similar to echogenicity of bone), defect size measured at the level of insertion of the umbilical cord, presence of gastric, bladder, or liver herniation, whether the majority of the bowel was out of the abdomen, amniotic fluid index, and the fetal AC and percentile.	IAID at a GA of 32 weeks, there was a 1.2 (95% CI 1.05-1.36, $p = 0.04$) greater odds of complex gastroschisis	NR

Fisher et al., 2020	Complex gastroschisis was diagnosed based on clinical determination of an atresia, perforation, or both present in the bowel at birth.	The first prenatal US was generally in the second trimester (median EGA 20 weeks [IQR 17.5-22.5 weeks]), and we defined this study as "second trimester" for the remainder fo the manuscript. Similarly, the final prenatal US was generally in the third trimester (35 weeks [IQR 33.5-36.5 weeks]) and was defined as such for consistency in the manuscript.	Prenatal ultrasound<br><br>Sonographic PACS software measuring tools (GE Viewpoint)	Bowel wall dilation (internal and external), bowel wall edema, and amount of external bowel (small (<40mm2), moderate(40-70mm2), or large(>70mm2)).<br>The "area of external defect" was defined as the point of ventral wall defect to the farthest extending loop of bowel in the horizontal plane, and from cranial to caudal extension in the vertical plane. Additional prenatal information included quantity of amniotic fluid (normal, polyhydramnios or oligohydramnios), as well as estimated fetal weight (EFW) determined from abdominal circumference (AC) for gestational age at time of scan.<br>Polyhydramnios	Polyhydramnios in third trimester US as a strong predictor of complex gastroschisis at birth ($p=0.011$), and several other ultrasound factors, including a large amount of external bowel on third trimester US, and an increase in bowel edema from second to third trimester US, with a high specificity and negative predictive value	NR
Geslin et al 2017	Complicated gastroschisis, characterized by gastrointestinal complications for 42 patients or by closing or vanishing gastroschisis (VG) for 10 patients.	Ultrasound was selected per trimester. For the first trimester (T1), it was the closest to 12 weeks of amenorrhea (WA), never less than 9 WA. For the second (T2) and the third (T3) trimesters, it was the closest to 22 +/-3 WA and 32 +/-3 WA, respectively	NR	Stomach dilatation IABD and EABD changes in bowel appearance such as hyperechoic bowel, segmental loss or increased peristalsis, and bowel wall thickening.<br>Bowel was qualitatively assessed as dilated when the internal bowel diameter (from inner wall to inner wall) was greater than 6 mm.	IABD and a small abdominal wall defect diameter accurately predict complex gastroschisis, and could be a first sign of vanishing gastroschisis when they occur early.<br>($p = 0.0003$)	NR
Marinović et al., 2018	Intestinal atresia, perforation or intestinal necrosis or strictures), in fetuses with gastroschisis with and without evidence of bowel dilatation.	NR	Prenatal ultrasound	IABD diameter (18 mm)<br>EABD diameter (18 mm)	IABD 11/15 (73%) ($p \geq 0.05$)<br>EABD 12/15 (82%) ($p = 0.91$)	NR
Martillotti et al., 2016	Any of the following complications within the first 10 days of life: atresia, perforation, necrosis, volvulus	Prenatal ultrasound markers were examined both at the end of the second and the third trimester of pregnancy.	Prenatal ultrasound	Intrauterine growth restriction<br>Intra-abdominal bowel dilatation (IABD) adjusted for gestational age, extra-abdominal bowel dilatation (EABD) ≥25 mm	IABD ($p = 0.001$)	In the absence of complications, vaginal delivery is planned at 38 weeks of gestation

		Performed monthly during the second trimester and every 2 weeks starting at 28–30 weeks. Fetal nonstress test monitoring is typically initiated at 32 weeks' gestation on a weekly basis.		Stomach dilatation<br>Stomach herniation<br>Perturbed mesenteric circulation<br>Absence of bowel lumen and echogenic dilated bowel loops (EDBL)		
Hijkoop et al., 2019	Complex gastroschisis was defined as gastroschisis complicated by intestinal atresia, volvulus, perforation and/or necrosis at primary evaluation at birth.	NR	2D ultrasound and Longitudinal 3D ultrasound measurements	Fetal stomach volume<br>Stomach-bladder distance	Fetal stomach volume and stomach-bladder distance, measured during pregnancy using 3D ultrasonography, do not predict complex gastroschisis.	NR
Hijkoop et al., 2017	NR	Following the diagnosis, several prenatal characteristics were assessed every 4 weeks. From 2007 onwards, additional assessments were scheduled weekly, starting at 30 weeks' gestation.	Prenatal ultrasound	Amniotic fluid index, considered abnormal if <5 cm (oligohydramnios) or >24 cm (polyhydramnios); intrauterine growth restriction, defined as estimated fetal weight ≤10th percentile for GA according to the Hadlock formula III14; IABD and extra-abdominal bowel dilatation (EABD) determined using a GA-specific nomogram, considering the bowel dilated if ≥13 mm at a GA of 25–30 weeks, ≥16 mm at 30–35 weeks, and if ≥26 mm at 35–40 weeks15; and intra-abdominal gastric dilatation, defined as measurements exceeding two SDs above the mean reference value, adjusted for GA.	Ultrasound markers could not reliably distinguish between simple and complex gastroschisis.	Vaginal delivery was planned from 37 weeks onwards, unless obstetric reasons required otherwise.
Kuleva et al., 2012	Intestinal atresia, stenosis, perforation, necrosis or volvulus	Starting from 30 weeks of gestation, routine follow up for fetuses with GS in our centre includes weekly ultrasound examinations (focusing on fetal growth, amniotic fluid volume and gastrointestinal appearance) integrated with daily fetal cardiotocography.	Prenatal ultrasound	Gestational age at examination, small-for-gestational- age (SGA) fetuses (estimated fetal weight <10th percentile on local fetal growth charts424–26), umbilical artery<br>Doppler abnormality (resistance index >95th centile), gastrointestinal appearance including echogenic bowel, thickened bowel (wall >3 mm), stomach	IABD (odds ratio 4.13, 95% CI 1.32–12.90;<br>P = 0.018), which also developed earlier in the complex GS group compared with the simple GS group (28.1 ± 3.6 versus 31.9 ± 3.9 weeks, P = 0.011).	In the absence of ominous finding at prenatal follow up, delivery is planned at 36–38 weeks of gestation, depending on fetal growth,

		Antenatal steroids are routinely administered at 30–32 weeks of gestation for acceleration of fetal lung maturation.		dilatation (anteroposterior or transverse diameters measured >2 SD from reference nomogram,27 stomach herniation, presence and degree of bowel dilatation (intra-abdominal or extra abdominal) and dilatation of multiple bowel loops.<br>Dilated loops of bowel were assessed quantitatively, considering intra-lumen diameter from inner wall to inner wall >6 mm as intra-abdominal or extra-abdominal bowel dilatation.	Moreover, each additional millimetre of dilatation was associated with a 10% increase in risk of complex GS (odds ratio 1.1, 95% CI 1.02–1.17; P = 0.015).	and obstetrician and paediatric surgeon availabilities. If an ominous fetal heart rate is found at cardiotocography, then emergency caesarean section is undertaken.
Robertson et al., 2017	Presence of coexisting intestinal anomalies at birth such as intestinal atresia, stenosis, perforation, necrosis or volvulus	NR	Prenatal ultrasound	EABD, intra-abdominal bowel dilatation, polyhydramnios, oligohydramnios, abnormal UA Dopplers, bowel matting, stomach herniation and dilatation were investigated as predictors.<br>Extra-abdominal bowel dilatation, intra-abdominal bowel dilatation, abnormal umbilical artery Dopplers (resistance index > 95th centile), gastric herniation, bowel matting, stomach dilatation (diameter greater than 95th centile for estimated gestational age), growth restriction (estimated fetal weight less than fifth centile), and abnormal amniotic fluid volume was collected from ultrasound image reports performed by MFM specialists.<br>Measurements of extra and IABD were further divided into three levels of dilatation: class 1 was 10–19 mm, class 2 was 20–29 mm and class 3 was equal to or greater than 30 mm	The presence of antenatal extra-abdominal bowel dilatation was shown to be statistically significant in predicting neonatal complex gastroschisis (P = 0.037).	NR
Lap et al., 2020	Atresia, volvulus, necrosis or perforation of the bowel at birth	Fetal anomaly scan at 18–22 weeks' gestation.<br>During each examination, fetal biometry and amniotic fluid index were evaluated and the	Ultrasound using a GE Voluson 730 or E8 (GE Healthcare,	Polyhydramnios was defined as an amniotic fluid index ≥24 cm17. Intra- and extra-abdominal bowel diameters were measured at the short axis of the bowel lumen (inner to inner wall) of the most	Cases with complex gastroschisis had larger intra- and extra-abdominal bowel diameters than did those	Delivery was planned from 37weeks onwards by induction of labor.

		pulsatility index (PI) of the umbilical artery (UA), SMA-PI and bowel diameter were measured.<br>All pregnancies with gastroschisis underwent ultrasound follow-up evaluations at 24, 28, 30, 32, 34, 35 and 36 weeks	Zipf, Austria) ultrasound machine, with a 4–8-MHz transabdominal transducer.	dilated bowel segment. Intra-abdominal SMA velocity measurements were obtained in a sagittal or axial plane of the fetal abdomen after its origin from the aorta, just above the renal arteries (with an angle of insonation below 30◦).<br>The extra-abdominal SMA was identified and its flow velocity measured directly distally to the abdominal wall defect.	with simple gastroschisis ($p<0.001$ and $p<0.005$, respectively)	
Nitzsche et al., 2020	Intestinal obstruction or atresia	After 30 weeks of pregnancy, ultrasound scans were performed weekly until delivery.	Prenatal ultrasound	The fetal growth, Doppler measurements, assessment of the amniotic fluid using the deepest pocket, the maximal IABD and EABD were assessed during the scans.<br>Using the saved images of the last scan between 30 to 34 weeks of pregnancy retrospectively, the same examiner performed the measurements of IABD and EABD.<br>Cut-offs of 10 mm for IABD and 18 mm for EABD were used.<br>Polyhydramnios was defined when the deepest single pocket measurement was above 8 cm.	EABD cut-off values of 10 mm and 18 mm showed low sensitivity and specificity to predict complex gastroschisis.	NR

AC: abdominal circumference; AWD: abdominal wall defect; EABD: extra-abdominal bowel dilatation; EBWT: extra-bowel wall thickness; IABD: intra-abdominal bowel dilatation; EAID: extra-abdominal intestinal diameter; IAID: intra-abdominal intestinal diameter.
